# Folding of a single domain protein entering the endoplasmic reticulum precedes disulfide formation

**DOI:** 10.1074/jbc.M117.780742

**Published:** 2017-03-15

**Authors:** Philip J. Robinson, Marie Anne Pringle, Cheryl A. Woolhead, Neil J. Bulleid

**Affiliations:** From the Institute of Molecular, Cell and Systems Biology, College of Medical, Veterinary and Life Sciences, University of Glasgow, Glasgow G12 8QQ, Scotland, United Kingdom

**Keywords:** disulfide, disulfide formation, endoplasmic reticulum, ER, protein disulfide isomerase, protein folding, protein translocation, β2-microglobulin

## Abstract

The relationship between protein synthesis, folding, and disulfide formation within the endoplasmic reticulum (ER) is poorly understood. Previous studies have suggested that pre-existing disulfide links are absolutely required to allow protein folding and, conversely, that protein folding occurs prior to disulfide formation. To address the question of what happens first within the ER, that is, protein folding or disulfide formation, we studied folding events at the early stages of polypeptide chain translocation into the mammalian ER using stalled translation intermediates. Our results demonstrate that polypeptide folding can occur without complete domain translocation. Protein disulfide isomerase (PDI) interacts with these early intermediates, but disulfide formation does not occur unless the entire sequence of the protein domain is translocated. This is the first evidence that folding of the polypeptide chain precedes disulfide formation within a cellular context and highlights key differences between protein folding in the ER and refolding of purified proteins.

## Introduction

Intramolecular disulfide bonds are a common feature of secretory proteins that can be crucial to structure, stability, and function (1). Disulfide bond formation requires the free thiol groups of two cysteine residues to be positioned in a correct steric conformation to enable thiol-disulfide exchange to occur with an accessible catalyst (2). This precise alignment of often widely separated cysteines is highly dependent on polypeptide chain folding. Once formed, disulfide bonds can influence further folding by constraining the polypeptide and restricting the number of possible folding conformations (3). Therefore, the process of disulfide formation is intimately coupled with folding (4), and the rules and mechanisms dictating this process remain a central question in protein folding research.

In the eukaryotic cell, proteins are synthesized on cytosolic ribosomes at a rate of ∼1–5 amino acids per second (5). This time span enables folding to occur in partially synthesized intermediates, before chain release. If a protein contains a signal sequence then it is targeted to the secretory pathway, where the extending nascent chain is extruded through the Sec translocon and into the endoplasmic reticulum (ER)^2^ (6). Here post-translational modifications, including signal peptide cleavage and glycosylation, can take place. During translocation the spatial restrictions within both the ribosome and Sec complex limit folding to chain compaction and formation of simple tertiary structure (7, 8). As the nascent chain emerges into the ER lumen, the increased space available allows for more complex structure formation (9, 10). In contrast to the cytosol, the ER environment is specialized for disulfide formation and rearrangements, with a carefully regulated redox balance to enable both oxidation and reduction of disulfides (11). These reactions are catalyzed by ER-resident protein disulfide isomerase (PDI) family members through the process of thiol-disulfide exchange (12). Although disulfide formation commonly occurs after chain release, it can occur in a growing chain, from the point where the second of two paired cysteine residues enter the ER (13). Despite experimental identification of intrachain disulfides in translation intermediates (14, 15), the relationship between the formation of disulfides and the processes of translocation and folding remains unclear.

In this study our aim was to characterize at what stage intrachain disulfides form in relation to polypeptide translocation, folding, and interactions with PDI. We designed a protein construct consisting of β2-microglobulin (β2M) with a C-terminal extension. β2M is a small (11 kDa) secreted protein that forms an immunoglobulin fold and contains a single intramolecular disulfide between its only two cysteine residues (16). The extension enables disulfide formation at the ER exposed N terminus, whereas the C terminus remains attached to the ribosome. This protein is expressed through *in vitro* translation reactions to generate stalled intermediates that are representative of the different stages of polypeptide translocation, providing insight into the early stages of protein compaction and disulfide formation.

## Results

### Experimental design

**Figure 1.**
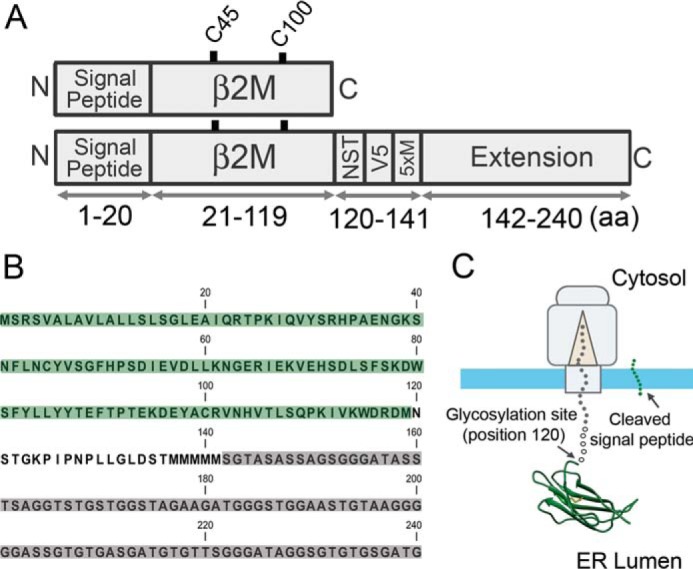
**Primary structure of the extended β2M construct and anticipated folding during translocation.** *A*, schematic representation of β2M with and without the extension showing the cysteine residues (Cys-45 and Cys-100) and the organization of key elements including the glycosylation site (NST), V5 epitope (V5), and methionine residues (Met_5_). *B*, the complete amino acid sequence of the extended β2M construct with the β2M domain highlighted in *green*, the additional elements (NST, V5, and Met_5_) in *white,* and the extension in *gray. C*, model of the ribosome-Sec complex displaying the extended β2M construct. The N-terminal β2M domain folds to the native structure once ER exposed, whereas the C terminus is attached to the cytosolic ribosome. *5xM*, Met_5_; *aa*, amino acid.

To provide a suitable substrate to study early folding events and disulfide formation using stalled translation intermediates, we added a C-terminal polypeptide extension onto human β2M (Fig. 1,*A* and *B*). This extension ensures that the β2M sequence will be fully translocated across the ER membrane whereas the polypeptide chain will still be attached to the ribosome (Fig. 1*C*). The extension provides an inert linker that should form a uniform extended structure between the folding domain and the ribosome, allowing control of ER exposure and clear interpretation of folding at the N terminus. Between β2M and the extension we engineered a glycosylation site (NST) to monitor translocation, a V5 epitope for immunoprecipitation, and a string of methionine residues (Met_5_) to boost the signal of the radiolabeled translation product.

The plasmid encoding extended β2M was used as PCR template for production of linear DNA fragments which were subsequently transcribed into RNA. The RNA was then used to program rabbit reticulocyte lysate translation reactions containing ER-derived dog pancreas microsomes (DPMs) or semipermeabilized cells (SP cells). The rabbit reticulocyte lysate does not contain added DTT and is, therefore, an appropriate system to study disulfide formation (17). Appropriate reverse primer design for the PCR (supplemental Table S3) enables linear DNA of any length to be produced so that the translation product can be controlled to the length of a single amino acid. Omitting a stop codon from the reverse primer generates templates encoding stalled translation intermediates. This system can be used to generate radiolabeled intermediates of any length to assess folding status and disulfide formation at defined stages of translocation. In addition to these stalled intermediates, we also generate transcripts that contain a stop codon to initiate release from the ribosome. We refer to the constructs without stop codons as stalled intermediates and those with stop codons as released.

### Glycosylation reveals the intermediate length required for full translocation of the globular domain

**Figure 2.**
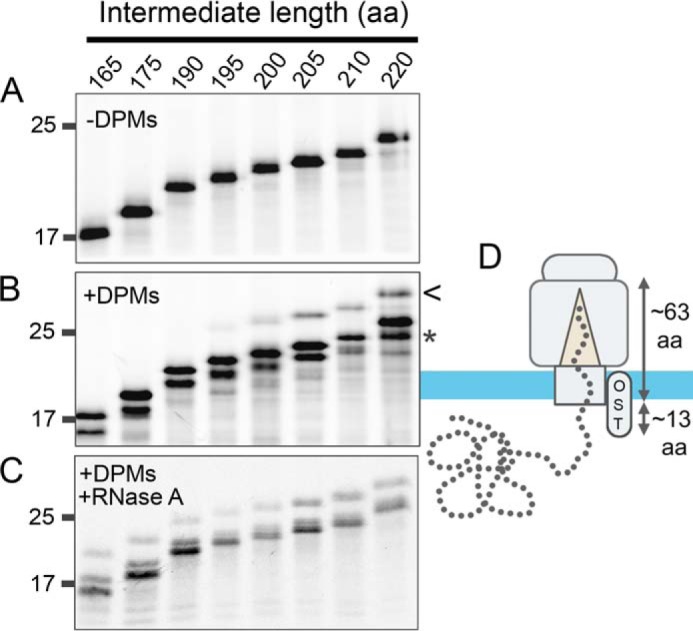
**Translation and translocation of extended β2M intermediates.** Autoradiographs of radiolabeled, immunoisolated translation product generated from stalled intermediates of between 165 and 220 amino acids. Gels were run under reducing conditions. *A* and *B*, translations were performed (*A*) in the absence of DPMs and (*B*) in the presence of DPMs. An example of signal peptide cleavage (*) and glycosylation (*arrow*) are highlighted for the 220 sample. *C*, the translation was performed in the presence of DPMs and treated with RNase A on completion. These data are representative of three independent repeats. *D*, model showing the approximate extension length required to span the ribosome-Sec complex as estimated from the glycosylation data.

To determine how folding events are coordinated in our stalled intermediates, we first estimated the chain length required to achieve full ER exposure of the β2M domain. The catalytic site of the oligosaccharide transferase complex (OST) is located on the luminal side of the ER membrane; therefore glycosylation (at position 120) is a useful assay for translocation. Stalled translation intermediates were purified by immunoisolation using a β2M-specific antibody and analyzed by reducing SDS-PAGE and autoradiography. In the absence of DPMs each translation product is present as a single band, which represents the unprocessed pre-protein (Fig. 2*A*). On addition of DPMs, targeting and translocation resulted in cleavage of the 20-amino acid signal peptide to produce the mature protein, which is detected at all chain lengths (Fig. 2*B, asterisk*). Glycosylation results in a band that runs higher than the translation product on SDS-PAGE and is present for intermediates 195 and longer (Fig. 2*B*). Therefore, we can estimate that it takes ∼75 amino acids (195 minus 120) to span the distance from the P site of the ribosome to the active site of the OST. It is known that it takes 12–14 residues of an extended peptide to reach the active site of the OST from the ER side of the membrane (18); therefore, we can estimate that it takes ∼63 amino acids from the P site of the ribosome before exposure to the ER lumen (Fig. 2*D*). Treatment with RNase A releases the translation product, improving the efficiency of signal peptide cleavage and exposing the glycosylation site, so that glycosylation is detected at all chain lengths (Fig. 2*C*). Importantly these results also demonstrate that the stalled translation intermediates are stably attached to the ribosome, as no glycosylation of intermediates of less than 190 amino acids occurs in the absence of added RNase.

### Disulfide formation only occurs once the full folding domain is exposed in the ER lumen

**Figure 3.**
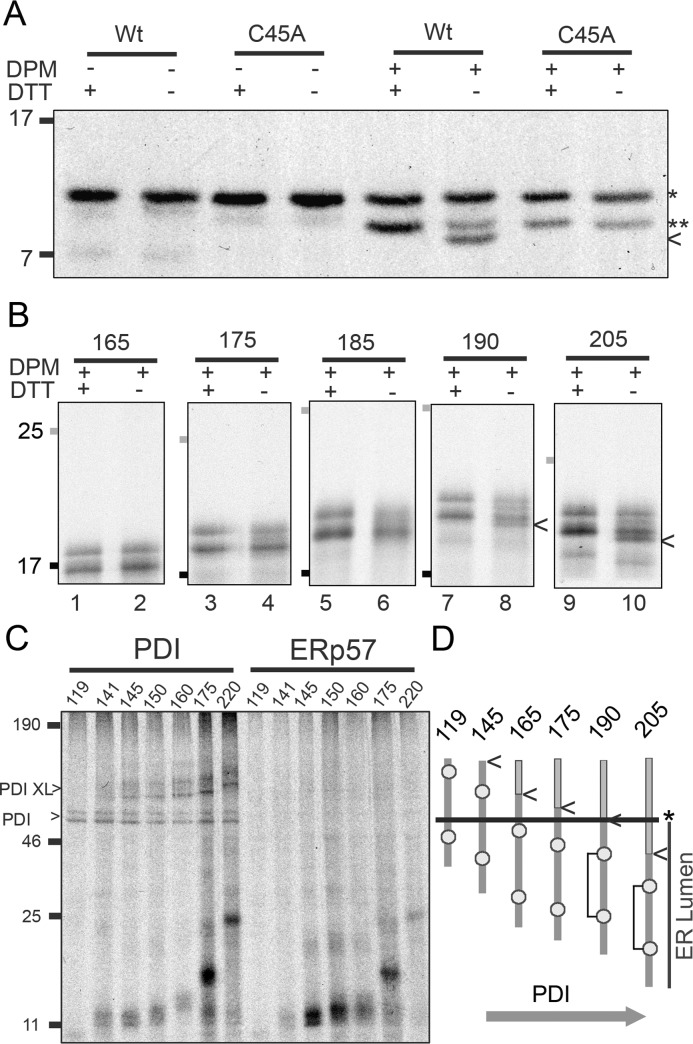
**Intrachain disulfide formation and PDI interactions.** Disulfide formation is assessed by SDS-PAGE analysis of immunoisolated, radiolabeled translation product under reducing (+DTT) and nonreducing (−DTT) gel conditions. *A*, autoradiographs show results for released, β2M (*Wt*, wild-type) and the single cysteine mutant (C45A) in the absence and presence of DPMs, with pre-protein (*) and mature protein (**) highlighted. Under nonreducing conditions oxidized β2M^Wt^ is detected by a shift in the mature protein (*arrow*). *B*, autoradiographs of stalled, extended β2M intermediates between 165 and 205 amino acids translated in the presence of DPMs. Molecular weight markers represent 17 (*black*) and 25 (*gray*). *Arrows* indicate the shift associated with disulfide formation for the 190 and 205 stalled intermediates under nonreducing conditions. Results shown are representative of five repeats (165–190 samples) and three repeats (205 sample). *C*, PDI interactions are detected through cysteine-specific cross-linking, followed by immunoisolation using a PDI antibody. Autoradiograph of a reducing gel shows a range of stalled intermediates (aa 119–220) with PDI-specific cross-links detected for intermediates from 141–220 (*PDI XL*). PDI expressed in the translation from endogenous RNA was also immunoisolated (*PDI*). The same samples were immunoisolated with an ERp57 antibody and no cross-links were detected. Data are representative of three independent repeats. *D*, diagram summarizing the timing of disulfide formation and PDI interactions relative to the entry of β2M into the ER. The *arrow* indicates the C-terminal end of the β2M sequence (position 120) and the *asterisk* shows the Sec-ER boundary.

The next step was to determine whether disulfide formation occurs in the stalled, ER-exposed translation intermediates. To assay disulfide formation we froze the disulfide status, on completion of translation, using an alkylating agent and compared nonreduced and reduced forms of the same samples on SDS-PAGE. To ensure that the single disulfide bond causes a detectable change in gel mobility, we first tested native released β2M (stop codon at position 120). In the absence of DPMs the pre-protein runs as a single band under both reducing and nonreduced conditions (Fig. 3*A*), demonstrating a lack of disulfide formation before targeting. On addition of DPMs the signal peptide is cleaved to produce the mature protein, which has a greater mobility (*arrow*) under nonreducing conditions indicating a disulfide. This change in mobility is absent from a mutant β2M (C45A), which contains a single cysteine residue and is, therefore, incapable of disulfide formation.

To assess disulfide formation in the extended β2M translation intermediates, we used a construct that does not contain a glycosylation site (β2M-extension AST) (supplemental Table S1) to simplify changes in electrophoretic mobility. According to the translocation data, ∼63 residues of the extension sequence are required to span the ribosome-Sec complex (Fig. 2*D*); therefore, the second of the two cysteine residues (position 100) is expected to enter the ER at an intermediate length of ∼165 residues, and disulfide formation could occur from this point onwards. Stalled intermediates were generated between 165 and 205 amino acids in length (Fig. 3*B*). At intermediate lengths between 165 and 185, the nonreduced samples do not shift in relation to the reduced samples (Fig. 3*B, lanes 1–6*) indicating an absence of disulfide formation. At intermediate lengths of 190 and 205 the presence of faster migrating species were detected in the nonreduced samples (*lanes 7–10*), revealing disulfide formation in these longer intermediates. Disulfide formation was also detected in the pre-protein as a band that runs between the pre-protein and mature protein. This indicates that disulfide formation can take place before signal peptide cleavage. The intermediate length required for disulfide formation (190 amino acids), coincides with the approximate ER exposure of the entire β2M sequence, which is 20 amino acids after the second of the two cysteine residues emerges. Therefore, despite both cysteine residues being exposed, disulfide formation does not occur until the whole of the β2M sequence is ER exposed.

### PDI interactions take place before disulfide formation

PDI is a highly abundant, ER-resident protein that catalyzes the process of disulfide formation (19) and can interact with nascent chains (20, 21). We used a cross-linking approach to determine whether PDI noncovalently interacts with extended β2M intermediates. For this purpose we added a cysteine-specific cross-linking agent (bismaleimidohexane) to stalled translation intermediates (β2M-extension AST) and then immunoisolated with a PDI antibody. Cross-linked complexes were detected between PDI and intermediates beginning at ∼145 amino acids (Fig. 3*C*) (a length at which we expect ER nascent chain exposure of ∼80 amino acids) and at all longer intermediate lengths. No cross-linking was seen to the 119 intermediate despite the availability of the first cysteine within the ER for cross-linking. The cross-linked products increase in size, reflecting the increase in length of the translation intermediates. Endogenous PDI translated and radiolabeled during the reactions was also detected by immunoisolation. PDI runs as two distinct bands, which reflect alternate conformations of the protein that are covalently stabilized by cross-linking, as reported previously (22). These bands are easily distinguished from cross-linked products as they migrate to the same distance in each sample (Fig. 3*C*). Control samples show no interaction with the PDI family member ERp57, which usually recognizes glycosylated substrates (23), demonstrating that the cross-linked products are specific to PDI. Endogenous radiolabeled ERp57 is absent probably because of a lower level of translation of the endogenous mRNA compared with PDI. The length of intermediates interacting with PDI indicates that it recognizes stalled intermediates where the second cysteine is not exposed to the ER lumen, *i.e.* before disulfide bond formation is possible (Fig. 3*D*).

### Refolding of β2M requires the disulfide bond

In the next set of experiments we describe folding status of a polypeptide chain. For clarity we refer to folding status, which reflects the extent to which the polypeptide has formed secondary and tertiary structures on its pathway to the native state. We also refer to compaction to reflect an ensemble of early folding intermediates that have undergone hydrophobic collapse.

**Figure 4.**
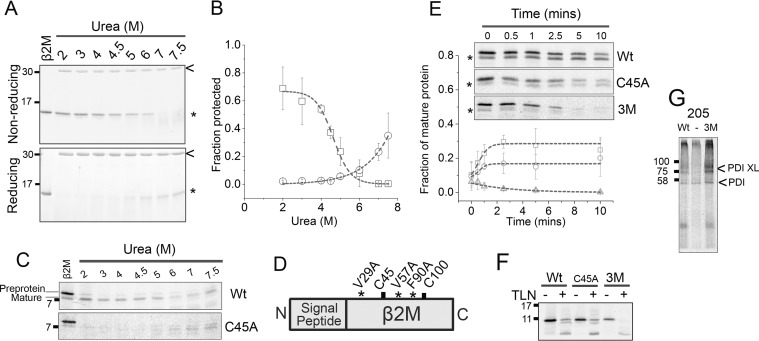
**The disulfide bond is not required for folding during native synthesis but stabilizes the final fold.** *A*, Coomassie Blue stained SDS-PAGE showing proteolysis of purified β2M (21–119) (*) by thermolysin (*arrow*) across a range of urea concentrations, under nonreducing and reducing conditions. *B*, refolding curves of reduced (*circles*) and oxidized (*squares*) β2M produced by quantification of gel bands following treatment (mean ± S.D. for *n* = 3). *C*, autoradiographs of thermolysin-induced proteolysis of refolded, released β2M (*Wt*) compared with the single cysteine mutant (*C45A*), produced through *in vitro* translation in the presence of DPMs. Data are representative of four independent experiments. *D*, schematic representation of β2M showing the location of the destabilizing mutations relative to the cysteine residues. *E*, proteolysis time courses of released β2M^Wt^ (*squares*), β2M^C45A^ (*circles*), and β2M^3M^ (*triangles*) produced through *in vitro* translation in the presence of DPMs. The *inset* shows representative autoradiographs of these time courses with the plot showing degradation of the mature protein (*) calculated from gel quantification of mature protein and subsequent normalization to total protein (pre-protein plus mature protein) (mean ± S.D. for *n* = 3). *F*, thermolysin (*TLN*) digestion of nondenatured, released β2M translation product (*Wt*, *C45A*, or *3M*) synthesized in the absence of DPMs. Image is representative of two independent experiments. *G*, PDI interactions detected by cross-linking to Wt or the 3M mutant, (−) = no template. Data are representative of three independent experiments. All gels in this figure were run under reducing conditions. Trend lines in *panels B* and *E* are for guidance only.

To evaluate the folding status of translocation intermediates we need a technique that can discriminate between unfolded and compact polypeptides. This can be done using protease susceptibility, in which typically the peptide backbone becomes protease protected on folding. To assess how protease resistance relates to the folding status of β2M, we investigated the refolding of purified recombinant protein (residues 21–119) following denaturation in urea (Fig. 4*A*). At high urea concentrations (6–7.5 M) the protein is highly susceptible to digestion. As the urea concentrations decrease the protein folds and we see the appearance of protease-resistant β2M. When the bands are quantified and plotted (Fig. 4*B*), the resulting refolding curve follows a cooperative two-state process with a midpoint of denaturation at ∼4.5 M urea. When the assay is repeated under reducing conditions the protein is protease susceptible even at low urea concentrations (2–5 M), indicating a failure to refold. At higher urea concentrations there is some undigested protein, which can be attributed to loss of thermolysin activity in high concentrations of urea and reducing agent. These agents destabilize thermolysin structure and cause reduction of catalytically and structurally important metal ions, which therefore results in loss of activity. Overall these experiments highlight the strict requirement for the disulfide to allow refolding of purified β2M from denaturant, a finding that agrees with previous studies (24).

We next adapted the proteolysis assay to assess protease resistance of β2M produced in the translation system. Saponin was added following completion of translation to permeabilize the membranes and allow thermolysin access to the ER lumen. We repeated the denaturant refolding experiment, comparing protease susceptibility of released (stop codon included at position 120) β2M or the C45A mutant (Fig. 4*C*). The mature protein refolded as evidenced by the presence of resistance bands across the range of urea concentrations. However, the C45A mutant failed to refold (no resistance), reflecting the same dependence on the disulfide bond as observed for purified β2M. Undigested protein is observed at high urea concentrations in both samples because of loss of thermolysin activity under these conditions.

### The disulfide bond is not required for protease resistance when β2M is synthesized in the translation system

To characterize the folding status of stalled translation intermediates requires proteolysis experiments in the absence of denaturant. Therefore, we needed an alternative control to represent the unfolded state of the protein. For this purpose we introduced three mutations (V29A, V57A, and F90A) (Fig. 4*D*) that are known to destabilize β2M (25) and compared the protease susceptibility of this triple mutant (3M) to the wild-type protein with a proteolysis time course. For these experiments we translated the released form of β2M (stop codon at position 120) in the presence of DPMs (Fig. 4*E*). For the wild-type protein there was a decrease in the amount of pre-protein and an increase in the amount of mature protein following protease digestion. Quantification of the mature protein (Fig. 4*E, plot*) shows that it remains stable over the 10-min time course, demonstrating the protease resistance of released wild-type β2M. The destabilizing mutations (3M) make both the pre-protein and the mature protein more protease susceptible, with both rapidly degraded on thermolysin treatment (Fig. 4*E, plot* and *inset*). When we tested the C45A mutant in the same assay (Fig. 4*E*), we expected the digestion profile to resemble the 3M mutant, because of the requirement for the disulfide for denaturant refolding. However, its protease sensitivity more closely resembled the wild-type protein. This result indicates a difference in folding status between the polypeptide synthesized in microsomes and that formed following refolding from denaturant.

Protease resistance can also be conferred by interactions with cellular proteins such as PDI. To investigate this possibility we assessed proteolytic digestion of translation products synthesized in the absence of DPMs and, therefore, PDI. We found the wild-type protein and the C45A mutant to be protease resistant but the 3M mutant to be protease susceptible (Fig. 4*F*). Proteins from the reticulocyte lysate could protect the nascent chain but it is highly unlikely that this would be selective for the wild-type protein and C45A mutant but not the 3M mutant. We also determined whether PDI binds to the 3M mutant. If resistance to proteolysis was merely a consequence of PDI binding then we would predict that PDI does not bind to the 3M mutant. We chose a construct of 205 amino acids as wild-type intermediates of similar length associated with PDI (Fig. 3*C*). We found that the wild-type construct as well as the 3M mutant cross-linked to PDI (Fig. 4*G*). Hence, binding of the 3M polypeptide to PDI does not protect the nascent chain from proteolysis. Therefore, these results support our interpretation that the protease resistance is conferred by folding.

### Compaction of the nascent chain occurs in intermediates partially exposed to the ER lumen

To assess protease resistance of the extended β2M intermediates we used SP cells derived from the human fibrosarcoma cell line HT1080 as an ER source, instead of DPMs. We switched to this system because it has been shown to result in more efficient folding of proteins in more native conditions, because of the presence of an intact ER (17). To confirm that translocation and disulfide formation were consistent with data gathered using DPMs, both the glycosylation and the oxidation experiments were repeated with similar results (supplemental Fig. S2). The only difference was the absence of disulfide formation in the pre-protein, suggesting more efficient signal peptide cleavage in the SP cell system. We also carried out translation under conditions that only labeled cysteine. This ensured an equal labeling of protease-resistant products as only the β2M coding region contains cysteine.

**Figure 5.**
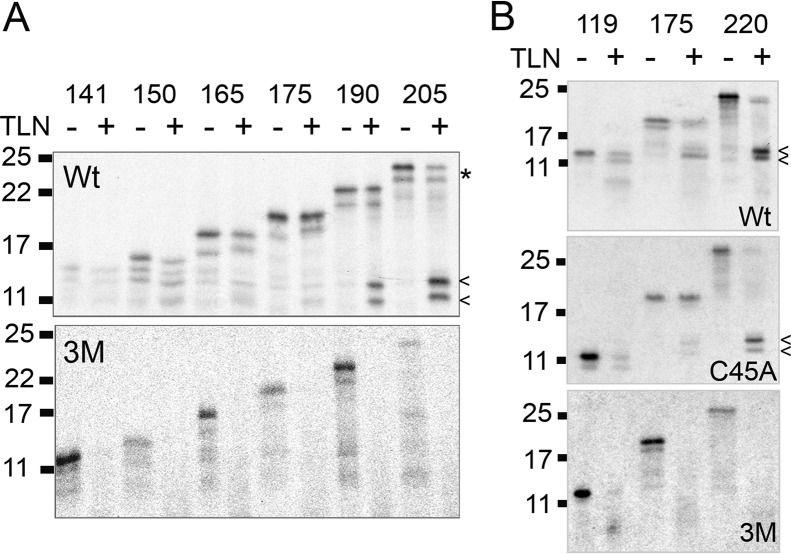
**Co-translational folding of extended β2M to protease resistant conformations proceeds as the nascent chain emerges.** Autoradiographs showing stalled translation intermediates of extended β2M produced in the presence of SP cells, assessed for protease susceptibility by thermolysin (*TLN*) treatment. *A* and *B*, digestion profiles of translation intermediates between (*A*) 141–205 and (*B*) 119–220 amino acids in length, for wild-type (*Wt*), 3M mutant (*3M*) and the single cysteine mutant (*C45A*). *Arrows* indicate lower molecular weight resistant bands and the *asterisk* highlights an example of undigested material for the 205 intermediate. The gels were run under reducing conditions and are representative of three or more independent experiments.

Stalled translation intermediates (β2M-extension AST 141–205) were assessed for protease susceptibility to determine their folding status (Fig. 5*A*). For each intermediate length tested, translation products remained after digestion, indicating that partially exposed intermediates compact to form protease-resistant conformations. This result with stalled intermediates in which the β2M domain is only partially exposed shows that compaction occurs before the second of the two cysteine residues is present in the ER lumen. Hence, the acquisition of protease resistance is independent of disulfide formation. When the length of the intermediate reaches 190, two prominent lower molecular weight bands are present (Fig. 5, *arrows*). This is the length at which the extension becomes ER exposed, indicating that protease susceptibility in this region causes release of protease-resistant β2M fragments (confirmed by further biochemical analysis (supplemental Fig. S3)). When the same experiment was repeated with the 3M mutant, all intermediates were completely digested, validating our interpretation that protease resistance reflects the folding status of the nascent chain.

Protease resistance was observed for the shortest stalled intermediate length (141), which has ∼78 residues of pre-protein exposed. To determine whether protease resistance occurs at even shorter stalled intermediates, we tested the 119 intermediate, in comparison to the 175 and 220 intermediates (Fig. 5*B*). We also included the C45A mutant to confirm our previous assumption that compaction occurs in the absence of a disulfide. A degree of resistance was detected in the shorter intermediate 119, indicating that compaction is occurring upon exposure of as few as 36 amino acids (119 − 63 − 20) of the mature sequence to the ER lumen. The C45A mutants gave rise to a similar digestion profile to that of the wild-type protein, confirming that the disulfide is not required for this initial compaction. All the 3M mutant intermediates tested were sensitive to protease digestion.

Taken together these proteolysis assays demonstrate that even short translation intermediates have protease resistance upon exposure to the ER lumen. This resistance indicates that nascent chain compaction can occur once sufficient polypeptide is exposed to the ER lumen (as long as there is time to do so), confirming that some folding occurs long before disulfide formation is possible. The complete sensitivity of the folding defective mutant validates the results with the wild-type sequence.

## Discussion

Previous efforts to evaluate the early stages of nascent chain folding have measured folding by either FRET- or NMR-based approaches and have used prokaryotic translation systems (26–28). The results from these studies have shown that folding can occur co-translationally with evidence of compact structures being formed by association with the ribosome. In this study we evaluated folding by the acquisition of protease resistance and crucially focused on events at the ER translocon. We showed that collapse of a polypeptide to a protease-resistant state occurred following exposure of less than 40 amino acids of the mature sequence to the ER lumen. Strikingly, a mutant polypeptide that is unable to reach the final native structure does not form the same protease-resistant state. Importantly, we have been able to demonstrate that the single disulfide does not form until the whole of the protein domain is translocated. In addition and in contrast to protein refolding, the disulfide is not required for compaction of the polypeptide chain to a protease-resistant state within the cellular milieu.

Previously, it has been shown that protein activity is achieved soon after the functional domain becomes exposed (10), requiring the full sequence to enter the ER lumen. In our system, compaction is observed with partial ER exposure, but disulfide formation, indicating fold completion, is delayed until β2M is fully exposed. These results suggest that formation of collapsed, protected conformations progresses incrementally as the nascent chain is extruded into the ER lumen but that the final native structure requires the entire β2M sequence to be available. This incremental progression parallels co-translational folding on prokaryotic ribosomes, in which compaction is observed within the vestibule of the ribosome, before native folding occurs on domain exposure (26). In other examples nascent chains remain completely unstructured until the full domain is exposed (29), some not achieving their fold until the domain is far past the ribosome (27).

On ER entry the nascent chain is exposed to resident folding factors including the disulfide catalyst PDI. This enzyme has been shown to interact with nascent chains directly to form intermolecular disulfides that are later resolved on disulfide formation (20). Our study provides insight into this interaction demonstrating PDI binding when the domain is partially ER exposed, before disulfide formation is possible. This result supports a previous study showing that PDI recognizes partially folded intermediates and remains bound throughout the folding process (30). Despite PDI binding at early stages, disulfide formation is delayed until the full β2M sequence is exposed, suggesting that the full sequence is required to form the tertiary structure to accurately position cysteines. The idea that folding drives disulfide formation is emerging as a common mechanism in proteins and is supported by findings from molecular dynamic simulations (31) and single molecule atomic force microscopy experiments (32), which both show that compaction and folding of the polypeptide chain precedes disulfide formation.

Our results also shed light on the function of the β2M disulfide bond, which is a highly conserved feature of the immunoglobulin fold (33). It has been established that the β2M disulfide is required for successful refolding from denaturant (24, 34), however, our results here indicate that this does not reflect folding *in vivo*, where the vectorial nature of protein synthesis and cellular environment allow compaction before disulfide formation. The results suggest that the role of the disulfide is to stabilize the folded state rather than initiate the attainment of the fold. This conclusion is supported by the finding that folding of immunoglobulin domains without disulfides can be restored through stabilizing mutations (35) and, although rare, functional immunoglobulin domains can exist without disulfides (36).

The model protein for this study was chosen because of its single domain and disulfide but it is also representative of the immunoglobulin family, which is often a modular component of large proteins, such as antibody chains. In such examples, domain exposure and opportunities for folding often occur before ribosome release. Therefore, the folding events described for the extended β2M construct are representative of immunoglobulin domains. Although the secreted proteins are diverse, mechanisms of co-translational oxidative folding are likely to share common features and, therefore, our results with this simplified experimental system are likely to be applicable to other single domain proteins with more complex disulfides. A bigger challenge will be to determine when native disulfide formation occurs between different protein domains in multidomain proteins such as the low density lipoprotein receptor (37). In this case, acquisition of the final native structure occurs post-translationally; non-native disulfides form prior to correct folding and are an obligatory requirement for correct folding. Our results go some way to address initial folding events but many questions remain to be answered before we obtain a clear picture of how proteins fold in the complex environment of the ER.

## Experimental procedures

### Molecular graphics

β2M (Fig. 1) was drawn using PDB file 1A1M using the UCSF Chimera package from the Resource for Biocomputing, Visualization, and Informatics at the University of California, San Francisco (38).

### Template generation

The β2M-extension construct was codon optimized and synthesized as plasmid DNA using GeneArt^TM^ gene synthesis (ThermoFisher Scientific) (DNA sequence, supplemental Fig. S1). Mutations were introduced by site-directed mutagenesis using appropriate primer pairs and ACCUZYME^TM^ (Bioline). The original plasmids or associated mutants (supplemental Table S1) were used as a template for PCR using a forward primer containing the T7 promoter, Kozak consensus sequence, and start of the β2M sequence (β2M-extension Fwd, supplemental Table S2). For stalled substrates we used various reverse primers that complement sequences corresponding to the extension region of the protein and that lack a stop codon (supplemental Table S3). Substrates that were released were produced using a reverse primer that contained a stop codon (supplemental Table S3). PCR products were ethanol precipitated, resuspended in nuclease-free water, and transcribed into RNA templates using T7 RNA polymerase (37 °C, 2 h) as described (23). The resulting RNA was also ethanol precipitated and suspended in nuclease-free water for use as template in translation reactions.

### Cell-free translation

DPMs were isolated from canine pancreas as described previously (39), snap-frozen, and stored at −80 °C. SP HT1080 human fibrosarcoma cells were prepared as described previously (17), and cells were added to a concentration of ∼10^5^ per 25 μl translation. Translations were performed using the Flexi^®^ Rabbit Reticulocyte Lysate System (Promega) containing amino acids minus methionine (20 μm), KCl (40 mm), glucose-6-P (5 mm) and radiolabeled with EasyTag^TM^ EXPRESS^35^S Protein Labeling Mix (PerkinElmer) (1 μl/25-μl reaction). Samples were incubated at 30 °C for 10 min in the absence or presence of DPMs or SP cells to translate protein with radiolabeled methionine residues. On completion samples were returned to ice and cycloheximide (2 mm) was added followed by *N*-ethylmaleimide (NEM) (20 mm). If samples required treatment with RNase A (0.2 mg/ml, 5 min at 30 °C) then it was added before cycloheximide/NEM. For proteolysis reactions, translation product was labeled at cysteine residues by adding amino acids minus Cys (in place of amino acids minus Met) and an excess of methionine (100 μm). Samples were processed through centrifugation and immunoprecipitation, as detailed below, for analysis through SDS-PAGE and autoradiography.

### Sample processing and immunoprecipitation

For glycosylation experiments DPM-containing samples were centrifuged (16,000 × *g*, 10 min) to isolate the microsomes and remove untargeted material. For disulfide formation analysis and cross-linking experiments, the pelleted DPMs were further washed with 150 μl high-salt buffer (3 m potassium acetate, 20 mm HEPES, pH 7.5) followed by 100 μl of 20 mm HEPES, pH 7.5, to further remove untargeted material and improve the clarity of the data. Pellets from cross-linking experiments were resuspended directly in immunoprecipitation (IP) buffer (50 mm Tris, pH7.5, 1% (v/v) Triton X-100, 150 mm NaCl, 2 mm EDTA, 0.5 mm PMSF, and 0.02% (w/v) sodium azide). Pellets from glycosylation/disulfide analysis were suspended in 50 μl of SDS buffer (25 mm Tris, 200 mm glycine, 0.8% (w/v) SDS) before addition of 0.9 ml IP buffer. For samples without DPMs, IP buffer was added directly to the samples following cycloheximide/NEM treatment.

In all cases the processed samples in IP buffer were incubated with 0.5% (v/v) Protein A-Sepharose (Generon) for 30 min (4 °C) and then centrifuged (2000 × *g*, 5 min) to pre-clear the samples of nonspecific precipitate. The resulting supernatant was then incubated with Protein A-Sepharose (0.5% v/v) and an appropriate antibody at 4 °C overnight. This was the polyclonal rabbit anti-human β2M antibody (Dako) at 1/10,000 for all experiments except for the cross-linking experiments which used PDI or ERp57 antibodies (at 1/1000) and experiments using the anti-V5 antibody (Invitrogen) (used at 1/10,000), where specified. The following day samples were isolated by centrifugation (2000 × *g*, 5 min) and the pellets were then washed (three times with 1 ml of IP buffer and once with 100 μl of 20 mm HEPES, pH 7.5) before eluting the protein by boiling in reducing SDS-PAGE sample buffer. To detect disulfide formation, samples were eluted from beads using nonreducing SDS-PAGE sample buffer, the samples were then split and 10 mm DTT added to the reducing sample before boiling. To prevent diffusion of DTT between gel lanes, samples were treated with 100 mm NEM before loading. In all translation experiments samples were analyzed by SDS-PAGE and autoradiography.

### Cross-linking

Translations were carried out as described above but without NEM treatment. Following completion the cross-linking agent bismaleimidohexane was added to a concentration of 1 mm before incubation (10 min) on ice. The reactions were quenched with 5 mm DTT and prepared for immunoprecipitation (described above).

### Denaturant refolding and proteolysis of purified protein and translation product

Recombinant protein was expressed and purified from the plasmid pINK as described previously (40). A 55-μm stock of purified β2M was prepared in 8.5 m urea and then diluted to 12 μm across a range of urea concentrations (2–7.5 M) in buffer containing 20 mm HEPES, pH 7.5, and 10 mm CaCl_2_. For reduced samples DTT was added (10 mm) to the unfolded protein stock before dilution and then in all subsequent buffers throughout the experiment. Thermolysin (5 mg/ml) in 2.5 m NaCl was added to samples to a final concentration of 0.2 mg/ml. After 10 min at room temperature proteolysis was quenched by the addition of 12.5 mm EDTA. Samples were analyzed by reducing SDS-PAGE (18% (w/v)) and stained with colloidal Coomassie Blue. Gel bands were quantified using ImageJ software, and normalized to undigested protein, to estimate the fraction of protein protected at each urea concentration. The values were then plotted to obtain the folding transition curves.

For refolding analysis of translation product, translations were performed in the presence of DPMs. Following cycloheximide and NEM treatment, 11 μl of translation product was denatured to a final volume of 100 μl in 8.5 m urea. Samples were then diluted to a final volume of 425 μl over a range of urea concentrations (2–7.5 M) containing 20 mm HEPES, pH 7.5, 10 mm CaCl_2_, and 0.1% (w/v) saponin. Proteolysis was carried out as described for the pure protein above but with 0.02 mg/ml thermolysin. After quenching with EDTA (75 mm) samples were directly resuspended in IP buffer before immunoisolation (β2M antibody) and analysis by reducing SDS-PAGE and autoradiography.

### Proteolysis of nondenatured translation product

Translations were carried out in the presence of DPMs (time courses in Fig. 4*E*) or SP cells (translation intermediates in Fig. 5 and supplemental Fig. S3) as described above, unless otherwise stated. Following cycloheximide, NEM, and saponin (0.1% (w/v)) treatment, thermolysin (0.02 mg/ml) and CaCl_2_ (10 mm) were added to initiate proteolysis. For time courses, aliquots were removed and quenched with excess EDTA at specific time points. Samples were immunoisolated and analyzed by reducing SDS-PAGE and autoradiography as described above. Bands were quantified using ImageJ and the values normalized to total protein (pre-protein plus mature protein) at the start of the reaction (0 min). For proteolysis analysis of intermediates, reactions were split two-thirds for digestion with the remaining one-third used as an undigested control sample for comparison. After 10 min, samples were quenched with EDTA, resuspended in IP buffer (1 ml) and immunoisolated. Proteolysis of samples translated in the absence of DPMs or SP cells were carried out in the same way but without saponin.

## Author contributions

P. J. R., C. A. W., and N. J. B. designed the experiments and wrote the paper. P. J. R. performed the experiments with assistance from M. A. P.

## Supplementary Material

Supplemental Data

## Footnotes

2 The abbreviations used are:

ER: endoplasmic reticulum
PDI: protein disulfide isomerase
β2M: β2-microglobulin
DPMs: dog pancreas microsomes
IP: immunoprecipitation
NEM: *N*-ethylmaleimide
OST: oligosaccharide transferase
SP: semipermeabilized
aa: amino acids.

## References

[1] Fass D. (2012) Disulfide bonding in protein biophysics. Annu. Rev. Biophys. 41, 63–79 22224600 10.1146/annurev-biophys-050511-102321

[2] Hudson D. A., Gannon S. A., and Thorpe C. (2015) Oxidative protein folding: from thiol-disulfide exchange reactions to the redox poise of the endoplasmic reticulum. Free Radic. Biol. Med. 80, 171–182 25091901 10.1016/j.freeradbiomed.2014.07.037 PMC4312752

[3] Camacho C. J., and Thirumalai D. (1995) Modeling the role of disulfide bonds in protein folding: entropic barriers and pathways. Proteins 22, 27–40 7675784 10.1002/prot.340220105

[4] Wallis A. K., and Freedman R. B. (2013) Assisting oxidative protein folding: how do protein disulphide-isomerases couple conformational and chemical processes in protein folding? Top. Curr. Chem. 328, 1–34 21630134 10.1007/128_2011_171

[5] Zhang G., and Ignatova Z. (2011) Folding at the birth of the nascent chain: coordinating translation with co-translational folding. Curr. Opin. Struct. Biol. 21, 25–31 21111607 10.1016/j.sbi.2010.10.008

[6] Pfeffer S., Dudek J., Zimmermann R., and Förster F. (2016) Organization of the native ribosome-translocon complex at the mammalian endoplasmic reticulum membrane. Biochim. Biophys. Acta 1860, 2122–2129 27373685 10.1016/j.bbagen.2016.06.024

[7] Woolhead C. A., McCormick P. J., and Johnson A. E. (2004) Nascent membrane and secretory proteins differ in FRET-detected folding far inside the ribosome and in their exposure to ribosomal proteins. Cell 116, 725–736 15006354 10.1016/s0092-8674(04)00169-2

[8] Nilsson O. B., Hedman R., Marino J., Wickles S., Bischoff L., Johansson M., Müller-Lucks A., Trovato F., Puglisi J. D., O'Brien E. P., Beckmann R., and von Heijne G. (2015) Cotranslational protein folding inside the ribosome exit tunnel. Cell Rep. 12, 1533–1540 26321634 10.1016/j.celrep.2015.07.065 PMC4571824

[9] Braakman I., and Bulleid N. J. (2011) Protein folding and modification in the mammalian endoplasmic reticulum. Annu. Rev. Biochem. 80, 71–99 21495850 10.1146/annurev-biochem-062209-093836

[10] Kowarik M., Küng S., Martoglio B., and Helenius A. (2002) Protein folding during cotranslational translocation in the endoplasmic reticulum. Mol. Cell 10, 769–778 12419221 10.1016/s1097-2765(02)00685-8

[11] Ellgaard L. (2004) Catalysis of disulphide bond formation in the endoplasmic reticulum. Biochem. Soc. Trans. 32, 663–667 15493982 10.1042/BST0320663

[12] Bulleid N. J. (2012) Disulfide bond formation in the mammalian endoplasmic reticulum. Cold Spring Harb. Perspect. Biol. 4, a013219 23125019 10.1101/cshperspect.a013219 PMC3536336

[13] Chen W., Helenius J., Braakman I., and Helenius A. (1995) Cotranslational folding and calnexin binding during glycoprotein synthesis. Proc. Natl. Acad. Sci. U.S.A. 92, 6229–6233 7541532 10.1073/pnas.92.14.6229 PMC41491

[14] Bergman L. W., and Kuehl W. M. (1979) Formation of an intrachain disulfide bond on nascent immunoglobulin light chains. J. Biol. Chem. 254, 8869–8876 113402

[15] Braakman I., Hoover-Litty H., Wagner K. R., and Helenius A. (1991) Folding of influenza hemagglutinin in the endoplasmic reticulum. J. Cell Biol. 114, 401–411 1650370 10.1083/jcb.114.3.401 PMC2289100

[16] Bjorkman P. J., Saper M. A., Samraoui B., Bennett W. S., Strominger J. L., and Wiley D. C. (1987) Structure of the human class I histocompatibility antigen, HLA-A2. Nature 329, 506–512 3309677 10.1038/329506a0

[17] Wilson R., Allen A. J., Oliver J., Brookman J. L., High S., and Bulleid N. J. (1995) The translocation, folding, assembly and redox-dependent degradation of secretory and membrane proteins in semi-permeabilized mammalian cells. Biochem. J. 307, 679–687 7741697 10.1042/bj3070679 PMC1136705

[18] Nilsson I. M., and von Heijne G. (1993) Determination of the distance between the oligosaccharyltransferase active site and the endoplasmic reticulum membrane. J. Biol. Chem. 268, 5798–5801 8449946

[19] Wilkinson B., and Gilbert H. F. (2004) Protein disulfide isomerase. Biochim. Biophys. Acta 1699, 35–44 15158710 10.1016/j.bbapap.2004.02.017

[20] Molinari M., and Helenius A. (1999) Glycoproteins form mixed disulphides with oxidoreductases during folding in living cells. Nature 402, 90–93 10573423 10.1038/47062

[21] Klappa P., Freedman R. B., and Zimmermann R. (1995) Protein disulphide isomerase and a lumenal cyclophilin-type peptidyl prolyl *cis-trans* isomerase are in transient contact with secretory proteins during late stages of translocation. Eur. J. Biochem. 232, 755–764 7588713

[22] Hawkins H. C., de Nardi M., and Freedman R. B. (1991) Redox properties and cross-linking of the dithiol/disulphide active sites of mammalian protein disulphide-isomerase. Biochem. J. 275, 341–348 2025221 10.1042/bj2750341 PMC1150058

[23] Jessop C. E., Chakravarthi S., Garbi N., Hämmerling G. J., Lovell S., and Bulleid N. J. (2007) ERp57 is essential for efficient folding of glycoproteins sharing common structural domains. EMBO J. 26, 28–40 17170699 10.1038/sj.emboj.7601505 PMC1782378

[24] Smith D. P., and Radford S. E. (2001) Role of the single disulphide bond of β_2_-microglobulin in amyloidosis *in vitro*. Protein Sci. 10, 1775–1784 11514668 10.1110/ps.4901 PMC2253195

[25] Smith D. P., Jones S., Serpell L. C., Sunde M., and Radford S. E. (2003) A systematic investigation into the effect of protein destabilisation on β_2_-microglobulin amyloid formation. J. Mol. Biol. 330, 943–954 12860118 10.1016/s0022-2836(03)00687-9

[26] Holtkamp W., Kokic G., Jäger M., Mittelstaet J., Komar A. A., and Rodnina M. V. (2015) Cotranslational protein folding on the ribosome monitored in real time. Science 350, 1104–1107 26612953 10.1126/science.aad0344

[27] Cabrita L. D., Cassaignau A. M., Launay H. M., Waudby C. A., Wlodarski T., Camilloni C., Karyadi M. E., Robertson A. L., Wang X., Wentink A. S., Goodsell L. S., Woolhead C. A., Vendruscolo M., Dobson C. M., and Christodoulou J. (2016) A structural ensemble of a ribosome-nascent chain complex during cotranslational protein folding. Nat. Struct. Mol. Biol. 23, 278–285 26926436 10.1038/nsmb.3182 PMC5405865

[28] Kim S. J., Yoon J. S., Shishido H., Yang Z., Rooney L. A., Barral J. M., and Skach W. R. (2015) Protein folding. Translational tuning optimizes nascent protein folding in cells. Science 348, 444–448 25908822 10.1126/science.aaa3974

[29] Eichmann C., Preissler S., Riek R., and Deuerling E. (2010) Cotranslational structure acquisition of nascent polypeptides monitored by NMR spectroscopy. Proc. Natl. Acad. Sci. U.S.A. 107, 9111–9116 20439768 10.1073/pnas.0914300107 PMC2889043

[30] Irvine A. G., Wallis A. K., Sanghera N., Rowe M. L., Ruddock L. W., Howard M. J., Williamson R. A., Blindauer C. A., and Freedman R. B. (2014) Protein disulfide-isomerase interacts with a substrate protein at all stages along its folding pathway. PLoS One 9, e82511 24465374 10.1371/journal.pone.0082511 PMC3896340

[31] Qin M., Wang W., and Thirumalai D. (2015) Protein folding guides disulfide bond formation. Proc. Natl. Acad. Sci. U.S.A. 112, 11241–11246 26297249 10.1073/pnas.1503909112 PMC4568676

[32] Kosuri P., Alegre-Cebollada J., Feng J., Kaplan A., Inglés-Prieto A., Badilla C. L., Stockwell B. R., Sanchez-Ruiz J. M., Holmgren A., and Fernández J. M. (2012) Protein folding drives disulfide formation. Cell 151, 794–806 23141538 10.1016/j.cell.2012.09.036 PMC3506382

[33] Halaby D. M., Poupon A., and Mornon J. (1999) The immunoglobulin fold family: sequence analysis and 3D structure comparisons. Protein Eng. 12, 563–571 10436082 10.1093/protein/12.7.563

[34] Isenman D. E., Painter R. H., and Dorrington K. J. (1975) The structure and function of immunoglobulin domains: studies with beta-2-microglobulin on the role of the intrachain disulfide bond. Proc. Natl. Acad. Sci. U.S.A. 72, 548–552 47633 10.1073/pnas.72.2.548 PMC432350

[35] Frisch C., Kolmar H., Schmidt A., Kleemann G., Reinhardt A., Pohl E., Usón I., Schneider T. R., and Fritz H. J. (1996) Contribution of the intramolecular disulfide bridge to the folding stability of REI_v_, the variable domain of a human immunoglobulin κ light chain. Folding Des. 1, 431–440 10.1016/s1359-0278(96)00059-4 9080189

[36] Rudikoff S., and Pumphrey J. G. (1986) Functional antibody lacking a variable-region disulfide bridge. Proc. Natl. Acad. Sci. U.S.A. 83, 7875–7878 3094016 10.1073/pnas.83.20.7875 PMC386825

[37] Jansens A., van Duijn E., and Braakman I. (2002) Coordinated nonvectorial folding in a newly synthesized multidomain protein. Science 298, 2401–2403 12493918 10.1126/science.1078376

[38] Pettersen E. F., Goddard T. D., Huang C. C., Couch G. S., Greenblatt D. M., Meng E. C., and Ferrin T. E. (2004) UCSF Chimera—a visualization system for exploratory research and analysis. J. Comput. Chem. 25, 1605–1612 15264254 10.1002/jcc.20084

[39] Kaderbhai M. A., and Austen B. M. (1985) Studies on the formation of intrachain disulphide bonds in newly biosynthesised bovine prolactin. Role of protein-disulphide isomerase. Eur. J. Biochem. 153, 167–178 4065147 10.1111/j.1432-1033.1985.tb09283.x

[40] Kad N. M., Thomson N. H., Smith D. P., Smith D. A., and Radford S. E. (2001) β2-microglobulin and its deamidated variant, N17D form amyloid fibrils with a range of morphologies *in vitro*. J. Mol. Biol. 313, 559–571 11676539 10.1006/jmbi.2001.5071

