# Supplementary material for: Folding of a single domain protein entering the endoplasmic reticulum precedes disulfide formation
Source: J Biol Chem. 2017 Mar 15;292(17):6978–86. doi: 10.1074/jbc.M117.780742 (PMC5409466; doi:10.1074/jbc.M117.780742)
Supplement: Supplemental Data [file supp_292_17_6978__index.html]

Folding of a Single Domain Protein Entering the Endoplasmic Reticulum Precedes Disulfide Formation — Folding of a single domain protein entering the endoplasmic reticulum precedes disulfide formation — Protein folding and disulfide formation — Supplemental Data 

# Folding of a single domain protein entering the endoplasmic reticulum precedes disulfide formation

## Supplemental Data

- Supplemental data (.docx, 173 KB) - Supplementary tables anf figure
