# Supplementary material for: Folding of a single domain protein entering the endoplasmic reticulum precedes disulfide formation
Source: J Biol Chem. 2017 Mar 15;292(17):6978–86. doi: 10.1074/jbc.M117.780742 (PMC5409466; doi:10.1074/jbc.M117.780742)
Supplement: Supplemental Data [file 10.1074_M117.780742_jbc.M117.780742-1.docx]

**Folding of a Single Domain Protein Entering the Secretory Pathway Precedes Disulfide Formation**

Philip J. Robinson, Marie Anne Pringle, Cheryl A. Woolhead and Neil J. Bulleid

Table S1: Plasmid list

Table S2: Forward primers for transcription/translation template generation

Table S3: Reverse PCR primers to generate transcription/translation templates

Figure S1: DNA sequence of the extended β2M construct (β2M-extension)

Figure S2: Translocation and disulfide formation of extended β2M intermediates in SP cells.

Figure S3: Identification of protease resistant fragments detected following digestion of extended-β2M intermediates that are 190 amino acid residues and longer.

**Table S1: Plasmid list**

| **Plasmid** | **Mutations introduced to β2M-extension** |
| --- | --- |
| β2M-extension | **-** |
| β2M-extension AST | N120A |
| β2M-extension AST C45A | N120A, C45A |
| β2M-extension AST 3M | N120A, V29A, V57A and F90A |

**Table S2: Forward primers for transcription/translation template generation**

| β2M-extension Fwd | GATGGCTAATACGACTCACTATAGGGTCAGGCCACCATGAGCAGATCTGTGGCCCTGG |
| --- | --- |
| β2M-extension mature Fwd | GATGGCTAATACGACTCACTATAGGGTCAGGCCACCATGATCCAGCGGACCCCCAAGATCCAGGTGTA |

**Table S3: Reverse PCR primers to generate transcription/translation templates**

| Stalled/Released Protein | Length of encoded protein (aa) | Primer sequence |
| --- | --- | --- |
| Stalled | 119 | CATGTCCCGGTCCCACTTC |
|  | 141 | CATCATCATCATCATGGTGCTGTC |
|  | 145 | GGCGGTGCCGGACATCATCAT |
|  | 150 | AGCAGAGCTGGCGCT |
|  | 160 | AGAGCTGGCTGTGGCTC |
|  | 165 | GCCGCCAGCAGATGTAGAG |
|  | 175 | TGTAGATCCGCCTGTAGAGC |
|  | 185 | GATCCTCCGCCTGTTG |
|  | 190 | AGCTCCGCCAGTAGATCCT |
|  | 195 | TGTTCCAGTAGAGGCAGCTC |
|  | 200 | TCCGCCCCCAGCAGCT |
|  | 205 | AGAAGAAGCTCCGCCTCC |
|  | 210 | GCCTGTGCCTGTTCCAGAAGAAG |
|  | 220 | TGTTGTTCCGGTGCCTGTAG |
| Released | 119 + Stop Codon | TTACATGTCCCGGTCCCACTTC |

**Figure S1. DNA sequence of the extended β2M construct (β2M-extension)**

ATGAGCAGATCTGTGGCCCTGGCTGTGCTGGCCCTGCTGTCTCTGTCTGGCCTGGAAGCCATCCAGCGGACCCCCAAGATCCAGGTGTACAGCAGACACCCCGCCGAGAACGGCAAGAGCAACTTCCTGAACTGCTACGTGTCCGGCTTCCACCCCAGCGACATCGAGGTGGACCTGCTGAAGAACGGCGAGCGGATCGAGAAGGTGGAACACAGCGACCTGAGCTTCAGCAAGGACTGGTCCTTCTACCTGCTGTACTACACCGAGTTCACCCCCACCGAGAAGGACGAGTACGCCTGCAGAGTGAACCACGTGACCCTGAGCCAGCCCAAGATCGTGAAGTGGGACCGGGACATGAACAGCACCGGCAAGCCCATCCCCAACCCTCTGCTGGGCCTGGACAGCACCATGATGATGATGATGTCCGGCACCGCCAGCGCCAGCTCTGCTGGATCTGGCGGCGGAGCCACAGCCAGCTCTACATCTGCTGGCGGCACAAGCACCGGCTCTACAGGCGGATCTACAGCAGGCGCTGCTGGCGCAACAGGCGGAGGATCTACTGGCGGAGCTGCCTCTACTGGAACAGCTGCTGGGGGCGGAGGCGGAGCTTCTTCTGGAACAGGCACAGGCGCCAGCGGCGCTACAGGCACCGGAACAACATCTGGCGGAGGGGCTACAGCTGGCGGAAGCGGAACCGGAACAGGCTCTGGCGCTACTGGG





**Figure S2: Translocation and disulfide formation of extended β2M intermediates in SP cells.** Autoradiographs show radiolabelled translation product representing stalled translation intermediates. Translocation (A) is monitored by glycosylation comparing intermediates (175-200) in the absence and presence of SP cells and following RNase A induced release (reducing gels). Glycosylation is highlighted by a star for the 200 intermediate (*). Data are representative of 2 repeats. (B) Disulfide formation in translation intermediates 175-190 assessed through comparison of reduced (+DTT) and non-reduced (-DTT) samples on SDS-PAGE. Arrow (<) indicates the shift associated with disulfide formation. Data are representative of 3 repeats.





**Figure S3: Identification of protease resistant fragments detected following digestion of extended-β2M intermediates that are 190 amino acid residues and longer.** (A) Dependence of the lower molecular weight resistant bands (<) on linker accessibility for the 165 and 205 intermediates, before and after release through RNase A treatment. The ribosome protected extension (lane 2) becomes protease sensitive on ribosomal release (lane 4), whilst the exposed extension (lane 6 and 8) is protease sensitive before release. (B) Identifying the position of the cleavage site by assessing glycosylation (<) in the NST sample and the reactivity of the fragments with β2M and V5 antibodies (released samples). The results show that the fragments contain the glycosylation site but not the V5 epitope. (C) Digestion of mature protein (21-205) and preprotein (1-205) in the absence of cells in comparison to the pre-protein (1-205) in the presence of cells. The migration of the bands indicates that the 2 protease resistant fragments correspond to preprotein and mature protein. To produce the 21-205 construct, an alternative forward primer was used to produce a substrate that lacked a signal sequence and started from position 21 (Table S2: β2M-extension mature Fwd). (D) Schematic summarising the approximate position of cleavage sites that lead to the presence of the resistant bands, with the lower arrows indicating the region of the resistant fragments. All gels in this figure were run under reducing conditions, repeated twice, and representative data is shown.
